# Insulin Resistance is Associated with MCP1-Mediated Macrophage Accumulation in Skeletal Muscle in Mice and Humans

**DOI:** 10.1371/journal.pone.0110653

**Published:** 2014-10-22

**Authors:** David Patsouris, Jingwei-Ji Cao, Guillaume Vial, Amelie Bravard, Etienne Lefai, Annie Durand, Christine Durand, Marie-Agnés Chauvin, Fabienne Laugerette, Cyrille Debard, Marie-Caroline Michalski, Martine Laville, Hubert Vidal, Jennifer Rieusset

**Affiliations:** 1 Institut National de la santé et de la recherche médicale, Unité Mixte de Recherche 1060, Laboratoire CarMeN, Université Lyon 1, Faculté de Médecine Charles Merieux Lyon-Sud, Lyon, France; 2 Centre de Recherche en Nutrition Humaine, Rhône-Alpes, Center for European Nutrition, Safety and Health, Pierre- Bénite, France; 3 Hospices civils de Lyon, Service de Nutrition et Diabétologie, Pierre- Bénite, France; Tohoku University, Japan

## Abstract

Inflammation is now recognized as a major factor contributing to type 2 diabetes (T2D). However, while the mechanisms and consequences associated with white adipose tissue inflammation are well described, very little is known concerning the situation in skeletal muscle. The aim of this study was to investigate, *in vitro* and *in vivo*, how skeletal muscle inflammation develops and how in turn it modulates local and systemic insulin sensitivity in different mice models of T2D and in humans, focusing on the role of the chemokine MCP1. Here, we found that skeletal muscle inflammation and macrophage markers are increased and associated with insulin resistance in mice models and humans. In addition, we demonstrated that intra-muscular TNFα expression is exclusively restricted to the population of intramuscular leukocytes and that the chemokine MCP1 was associated with skeletal muscle inflammatory markers in these models. Furthermore, we demonstrated that exposure of C2C12 myotubes to palmitate elevated the production of the chemokine MCP1 and that the muscle-specific overexpression of MCP1 in transgenic mice induced the local recruitment of macrophages and altered local insulin sensitivity. Overall our study demonstrates that skeletal muscle inflammation is clearly increased in the context of T2D in each one of the models we investigated, which is likely consecutive to the lipotoxic environment generated by peripheral insulin resistance, further increasing MCP1 expression in muscle. Consequently, our results suggest that MCP1-mediated skeletal muscle macrophages recruitment plays a role in the etiology of T2D.

## Introduction

Type 2 diabetes (T2D) is a complex multifactorial disease afflicting an increasing number of patients worldwide. Obesity is tightly associated with the onset of the disease and as such its prevalence follows obesity figures. In the last decade, inflammation was demonstrated to play a causative role in the etiology of diabetes [1]–[4]. First, it was shown that inflammation is elevated in white adipose tissue (WAT) of obese patients, which was latter on attributed to the recruitment of pro-inflammatory macrophages [5]. Subsequent studies involving gain and loss of function experiments have clearly indicated that classically activated (M1) macrophages promote the onset of diabetes in rodents [6], [7]. This contribution of inflammation in promoting insulin resistance is not only observed in the WAT but also in other insulin target tissues such as liver and skeletal muscles. As such, ablation of the F4/80+CD11b+CD11c+, macrophages, which mediate at least some of the diabetogenic properties of fatty acids, improves insulin sensitivity at both systemic and skeletal muscle levels [6], [8]–[10]. While attenuating inflammation is associated with improvement of skeletal muscle insulin sensitivity, there is no clear information on the mechanisms involved. As infiltration of inflammatory cells (i.e. M1 macrophages) is largely established in WAT, one possible mechanism is the modulation of cytokine concentrations at the systemic level, which may affect skeletal muscle insulin signaling in an endocrine manner. However, this hypothesis is questionable since plasmatic concentrations of pro-inflammatory cytokines are generally very low (e.g. 0–5 pg/ml for TNFα) [2], [9], [11], [12] and recently, it was shown that larger molecules such as the TNFα inhibitor etanercept, fails to improve insulin sensitivity [2]. Alternatively, macrophages are found in non adipose tissues such as skeletal muscle of obese and diabetic mice and patients [8], [12]–[18]. In obese and diabetic rodents, muscle macrophages were both described between muscles fibers and around the adipose tissue infiltrating muscles, suggesting that local inflammation in the skeletal muscle could contribute to insulin resistance [19]. This hypothesis is further supported by a recent publication establishing that skeletal muscle anti-inflammatory macrophages correlate with insulin sensitivity in obese and type 2 diabetic patients [18] Furthermore, mice overexpressing the anti-inflammatory cytokine IL10 in skeletal muscle and mice full body knock-outs for MCP1 are protected from high-fat diet-induced macrophage infiltration in muscle and insulin resistance [15], [17], which highlights a close relationship between muscle inflammation and insulin sensitivity. Nevertheless, the characteristics of inflammation in rodents are so far restricted to the high fat diet-induced obese mice model [15], [17], and the exact phenotype of macrophages and the mechanisms governing the recruitment of these latter in the skeletal muscle remain poorly understood.

In the present study, we characterized local inflammation and skeletal muscle macrophage accumulation in different rodent models of insulin resistance (ob/ob mice and both high fat diet- and palm oil-enriched diet-fed mice) and in type 2 diabetic patients. As the expression of the chemokine MCP1 was systematically elevated in the skeletal muscle of all these models, we further investigated the role played by this chemokine in the recruitment of muscle macrophages and in the onset of insulin resistance. These questions were addressed in creating a transgenic mouse line specifically overexpressing MCP1 in skeletal muscle. We demonstrate that the specific overexpression of MCP1 in muscle increased local inflammation while attenuating local insulin signaling, which suggested a role of MCP1-mediated muscle macrophage recruitment in the etiology of T2D.

## Methods

### Chemicals and reagents

Collagenase, palmitic acid and low endotoxin, FFA free BSA were from Sigma (Saint-Quentin Fallavier, France). Rosiglitazone was from Molekula (Gillingham, UK).

### Human subjects

One group of 8 volunteers was age-matched with 9 non diabetic obese subjects and 10 obese, type 2 diabetic patients. The characteristics of these subjects are described in Table 1 and have already been presented in a previous study [1]. All participants gave their written consent after being informed of the nature, purpose and possible risk of the study. The protocol and consent procedure were approved by the ethical committees of the Hospices Civils de Lyon and performed according to French Legislation (Huriet Law). After an overnight fast, blood was collected and percutaneous biopsies of the *vastus lateralis* muscle were performed under local anesthesia [2].

**Table 1:** Metabolic characteristics of lean, obese and type 2 diabetic patients (n = 8–10) in fasting state.

	Control	Obese	NIDM
Sex (M/F)	6F/2M	7F/2M	7F/3M
Age (years)	45±1	46±2 (NS)	48±2(NS)
Weight (kg)	62.3±3.7	89.7±3.4 (**)	82.7±3.3 (‡)
BMI (kg/m^2^)	22.9±0.5	32.3±0.9 (**)	31.2±0.7 (‡)
TG (µmol/l)	838±136	1150±202 (NS)	1411±220 (NS)
Glycemia (mmo/l)	4.9±0.11	5.2±0.3 (NS)	11.4±1.1 (‡¥)
Insulin (pmol/l)	37.7±3.7	69.9±17.5 (NS)	59.8±10.1 (NS)
HOMA-IR	1.38±0.15	2.82±0.73 (NS)	4.56±0.57 (‡)
Free Fatty Acids (µmol/l)	588±59	457±46 (NS)	710±37 (¥)

** p<0.01 obese Vs control; p<0.01 NIDM Vs Control, ¥p<0.01 obese Vs NIDM; NS: Not Significant. (NIDM, Non Insulin-dependent Diabetes Mellitus).

### Animals

Mouse procedures were approved by the French national ethic committee (CNREEA). Male C57Bl6 wild-type (wt) and ob/ob mice were obtained from Janvier (St Berthevin, France). For the high fat diet (HFD) protocol, C57Bl6 male mice were from Charles River (France). HFD (D12492, 45% Kcal) was from Research Diets and rosiglitazone was mixed as powder to the diet (200 mg/kg) [3]. The nutritional intervention with Rapeseed, Sunflower and palm oils (20%) was previously described in details [4].

### Lentiviral transgenesis

Mouse MCP1 full lenght cDNA inserted in pCR4TOPO was purchased at Source Bioscience (Nottingham, UK). MCP1 cDNA was subsequently subcloned downstream of the mouse muscle Creatin Kinase promoter in the pBS MCK vector. MCK promoter and MCP1 cDNA were subsequently inserted in the FG12 plasmid. Lentiviral particles were produced and injected in C57Bl6 embryo by the human virology department of the Ecole Normale Supérieure of Lyon (France) [5]. Mice carrying a single copy of the transgene were subsequently selected and amplified.

### Metabolic studies

Ip glucose and insulin tolerance tests (IPGTT and IPITT, respectively) were performed on 6h-fasted mice. Mice were injected ip with 2 mg/g body weight of glucose or 0.75 mU/g body weight of insulin, and blood glycemia was measured at indicated times. *Ex vivo* insulin signalling assay was performed on freshly isolated gastrocnemius as previously described [6].

### Immunohistochemistry

F4/80 antibody (Acris antibodies, Herford, Germany) was applied to acetone-fixed cryostat sections of frozen tissue samples and revealed according to an indirect immunoperoxidase technique [7].

### CD45+ isolation

Gastrocnemius and quadriceps of mice were minced and digested in trypsin (0.05%). The resulting cells were then incubated with CD45 microbeads (Miltenyi Biotech, Paris, France) and passed on a separation column (Miltenyi Biotech). CD45- and CD45+ were then collected following manufacturer's instructions.

### Adenoviral preparation

MCP1 cDNA was subsequently subcloned into pcDNA3 vector (Invitrogen, Saint Aubin, France) prior to insertion in V4 adenovirus genome [6], [8]. Adenoviral particle were amplified in Hek293 cells and titered with adenovirus quicktiter immunoassay ELISA kit (Cell Biolabs, San Diego, USA).

### Cell culture and chemotaxis assay

Raw 264.7 (ATCC) were maintained in DMEM supplemented with 10% fetal-calf serum (FCS). C_2_C_12_ (ATCC) were maintained in DMEM supplemented with 10% FCS and differentiated for 7 days in DMEM/2% FCS. Migration assays were performed with C_2_C_12_ conditionned media prepared in absence of serum for 24 hours, 8 µM pore size transwells were used (Corning, Avon, France). Palmitate was suspended in BSA to a ratio of 5∶1 (mol/mol) [6]. Detailed procedure is described by Nguyen et al. [9].

### RNA quantification

RNA extraction and real-time RT-PCR procedure analysis have been described previously [1]. Primers were specificaly designed to amplify specific amplicons, and their sequences are available on Table S1 in File S1.

### Western-blotting

Proteins were extracted in RIPA buffer in presence of phosphatase and protease inhibitors. Proteins were then seperated on polyacrylamide gels (Invitrogen). Phospho Akt (Ser473) and Akt antibodies were from Cell Signaling 5St Quentin Yvelines, France). α-Tubulin antibody was from Santa Cruz (Heidelberg, Germany) [1].

### ELISA and Kits

All assays used in this section were used according to manufacturers recommendations. IL10, MCP1 and TNFα ELISA kit were from Eurobio AbCys (Courtaboeuf, France). Insulin ultra sensitive ELISA kit was from Alpco. Free Fatty Acids, half microtest was from Roche (Meylan, France) and triglycerides assay kit was from Biomerieux (Craponne, France).

### Statistical analysis

Data are presented as means ± SEM. Student's unpaired t-test were applied and a cut-off was set to p<0.05. For analyses on human biopsies and on the NCD, HFD±Rosi experiments, ANOVA followed by a PLSD Fisher post-hoc test was used to determine the differences between the groups. Correlation analyses were performed with GraphPad Prism software (La Jolla, USA).

## Results

### Inflammation is increased in skeletal muscle of ob/ob mice

**Figure 1:**
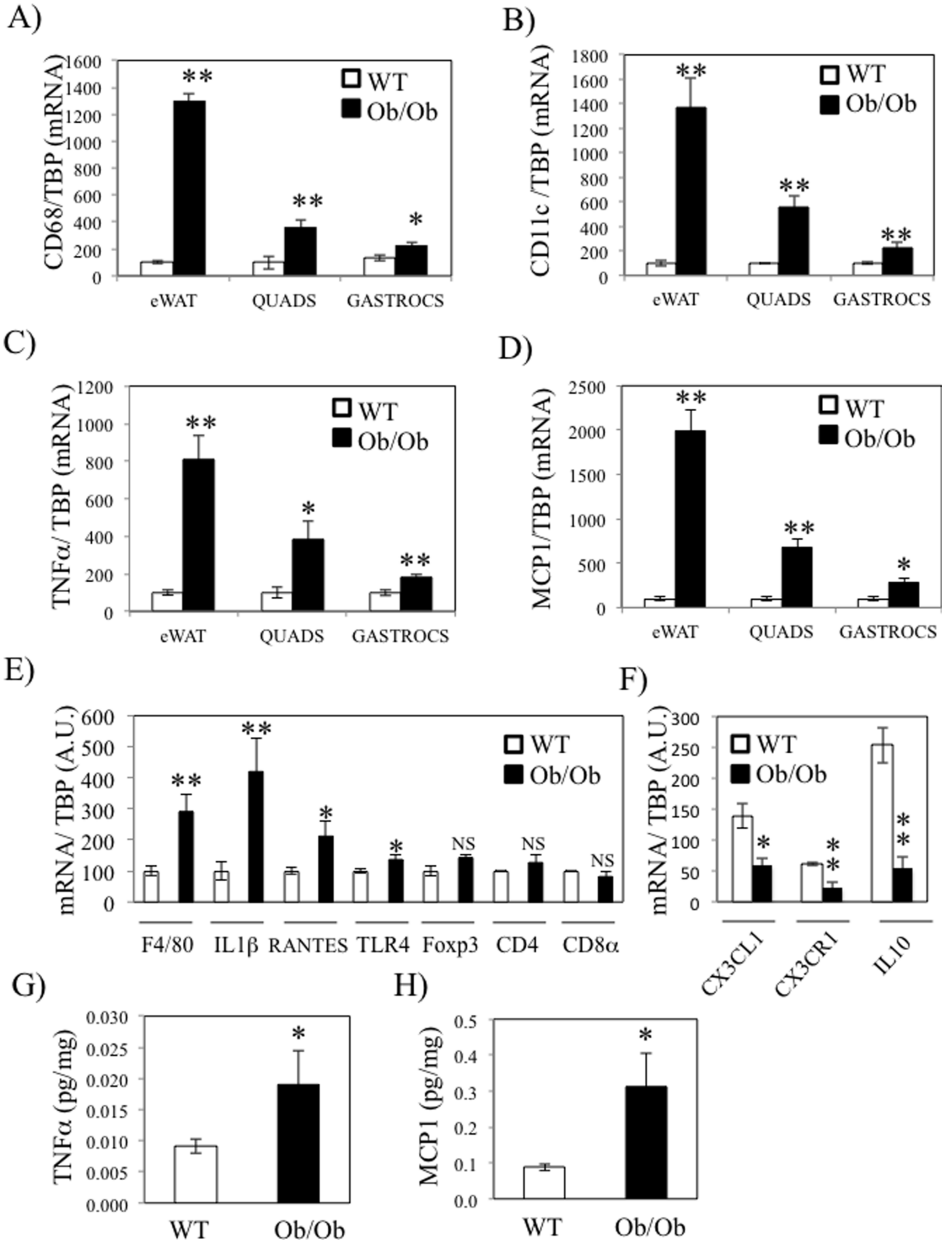
Skeletal muscle inflammation is increased in ob/ob mice. Epidydimal fat (eWAT), quadriceps (quads) and gastrocnemius (gastrocs) of wild –type (wt) and ob/ob mice were collected prior to analysis with qPCR of CD68 (A), CD11c (B), TNFα (C) and MCP1 D) qPCR analysis was performed for F4/80, IL1β, RANTES, TLR4, Foxp3, CD4 and CD8a in quadriceps from wt and ob/ob mice. E) qPCR analysis was performed for CX3CL1, CX3CR1 and IL10 in wt and ob/ob mice quadriceps. F–G) TNFα (E) and MCP1 (F) protein concentration in gastrocnemius of wt and ob/ob mice were quantified by ELISA. * stands for *P*<0.05 and ** for *P*<0.01; n = 5.

Muscle inflammation was firstly investigated in both gastrocnemius and quadriceps of genetically obese and insulin resistant mice (*ob/ob*). Mice physiological parameters are reported in Table S2 in File S1. Insulin resistance of *ob/ob* mice was illustrated by the significant increase of glycemia, insulinemia and of both blood and muscular TG (Table S2 in File S1). QPCR were performed in epididymal fat (eWAT), gastrocnemius and quadriceps in order to quantify the relative expression of the macrophage specific antigen CD68, the M1 macrophages specific marker CD11c, the pro-inflammatory cytokine TNFα and the macrophage chemokine MCP1 (CCL2). Consistent with other studies [10]–[13], the expression of all inflammatory markers was much more elevated in eWAT of *ob/ob* mice (10-fold) compared to wt mice (Figure 1A–D). This upregulation was also observed in quadriceps (4-fold) and gastrocnemius (2-fold) of ob/ob mice (Figure 1A–D). In addition, we measured other inflammatory markers, including lymphocytes specific markers, in quadriceps of wt and *ob/ob* mice. We found that the mRNA levels of the macrophage marker F4/80, the cytokine IL1β and the chemokine RANTES (CCL5) were also increased in the quadriceps of *ob/ob* mice compared to wt mice, whereas the lymphocytes specific markers Foxp3, CD4 and CD8 were not changed (Figure 1E). The higher expression of CD11c and of the pro-inflammatory cytokines TNFα and IL1β were supportive of the notion that macrophages within skeletal muscle of *ob/ob* mice were of the M1 phenotype. We therefore quantified the expression of CX3CR1, a marker of a subtype of anti-inflammatory macrophages, expressing high level of IL10 and responsive to the chemokine CX3CL1 (fractalkine) (Figure 1H) [14]. Interestingly, CX3CR1 expression was down regulated in parallel with the expression of CX3CL1 and IL10 in the skeletal muscle of *ob/ob* mice. We also quantified the protein concentration of TNFα and MCP1 in gastrocnemius and observed an increase of both proteins in *ob/ob* mice in agreement with RNA data (Figure 1G–H). Interestingly, TNFα concentration was also more elevated in plasma of *ob/ob* mice (Table S2 in File S1) and its estimated local intra muscular concentration was 50–100 more elevated than in plasma (400 pg/ml ±110 in *ob/ob* mice).

### Macrophage accumulation is increased in skeletal muscle of ob/ob mice

**Figure 2:**
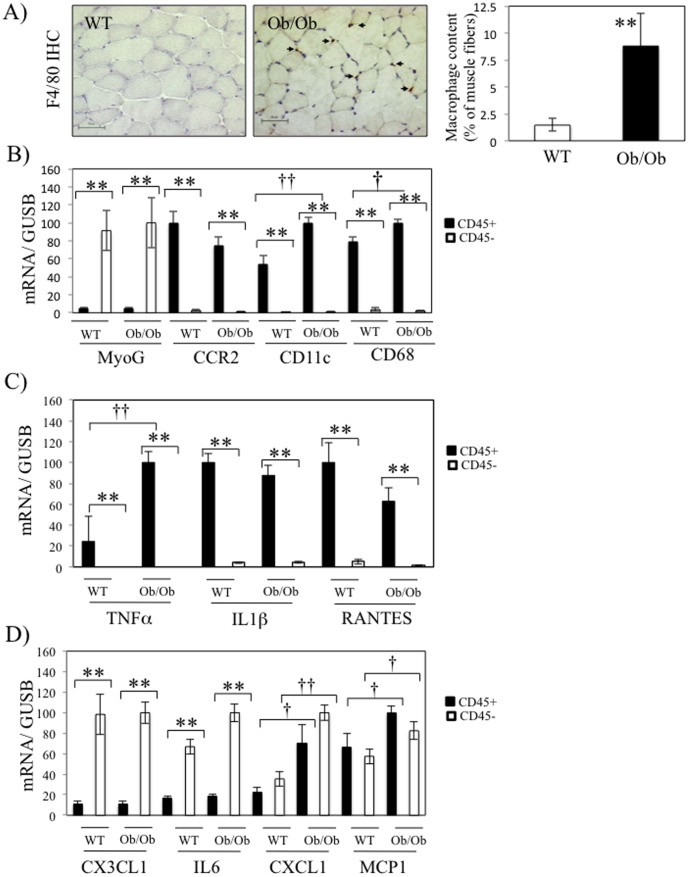
Macrophage recruitment in skeletal muscle of ob/ob mice. A) Representative images (left panel) and quantitative analysis (right panel) of the immunostaining with F4/80 antibody in gastrocnemius of wild-type (wt) and ob/ob mice. B–D) Following digestion of skeletal muscles (gastrocnemius and quadriceps), CD45+ and CD45- cells were purified and qPCR were performed on the indicated genes in wt and ob/ob mice. The different histograms regroup the genes for the validation of cell fractionation (B), the genes more expressed in CD45+ cells (C) or the genes either more expressed in CD45- cells or similarly expressed in both fractions (D).* stands for *P*<0.05 and ** for *P*<0.01 for the comparison between CD45- and CD45+ fractions, and † stands for *P*<0.05 and †† for *P*<0.01 for the comparison between WT and ob/ob mice; n = 5.

In order to better characterize inflammation within skeletal muscles of *ob/ob* mice, we performed an immuno-stainning of F4/80, a macrophage marker, on the quadriceps of mice. As shown in Figure 2A, F4/80 positive macrophages are significantly increased in muscle fibers of *ob/ob* mice compared to wt mice. In order to clarify in which cell population these markers were expressed, we digested quadriceps and gastrocnemius of wt and *ob/ob* mice and isolated the leukocytes population represented by the CD45+ cells. The CD45- cells represent all the other cells dislodged from the muscle, including the muscle fibers. As shown in Figure 2B, CD45+ cells did not express the muscle/myotube specific marker Myogenin (MyoG), whereas the macrophages markers (CD68, CD11c and CCR2) were barely detected in CD45- cells, which validated the subcellular fractionation. Interestingly, we observed that mRNA levels of TNFα, IL1β and RANTES were specificaly expressed in CD45+ cells (Figure 2C). Furthermore, TNFα and CD11c expression were more elevated in the fraction of CD45+ cells from *ob/ob* mice (Figure 2B and 2C), indicating that leukocytes are in a pro-inflammatory state in skeletal muscles of *ob/ob* mice compared to wt. On the other hand, the chemokine CX3CL1 and the cytokine IL6 were more abundantly expressed in the CD45- fractions (Figure 2D). Finally, the two chemokines CXCL1 and MCP1 expression were similarly detected in both CD45+ and CD45- cells and their expression were more elevated in *ob/ob* mice in both fractions (Figure 2D).

### Inflammation is associated with insulin sensitivity in skeletal muscle of diet-induced obese and diabetic mice

**Figure 3:**
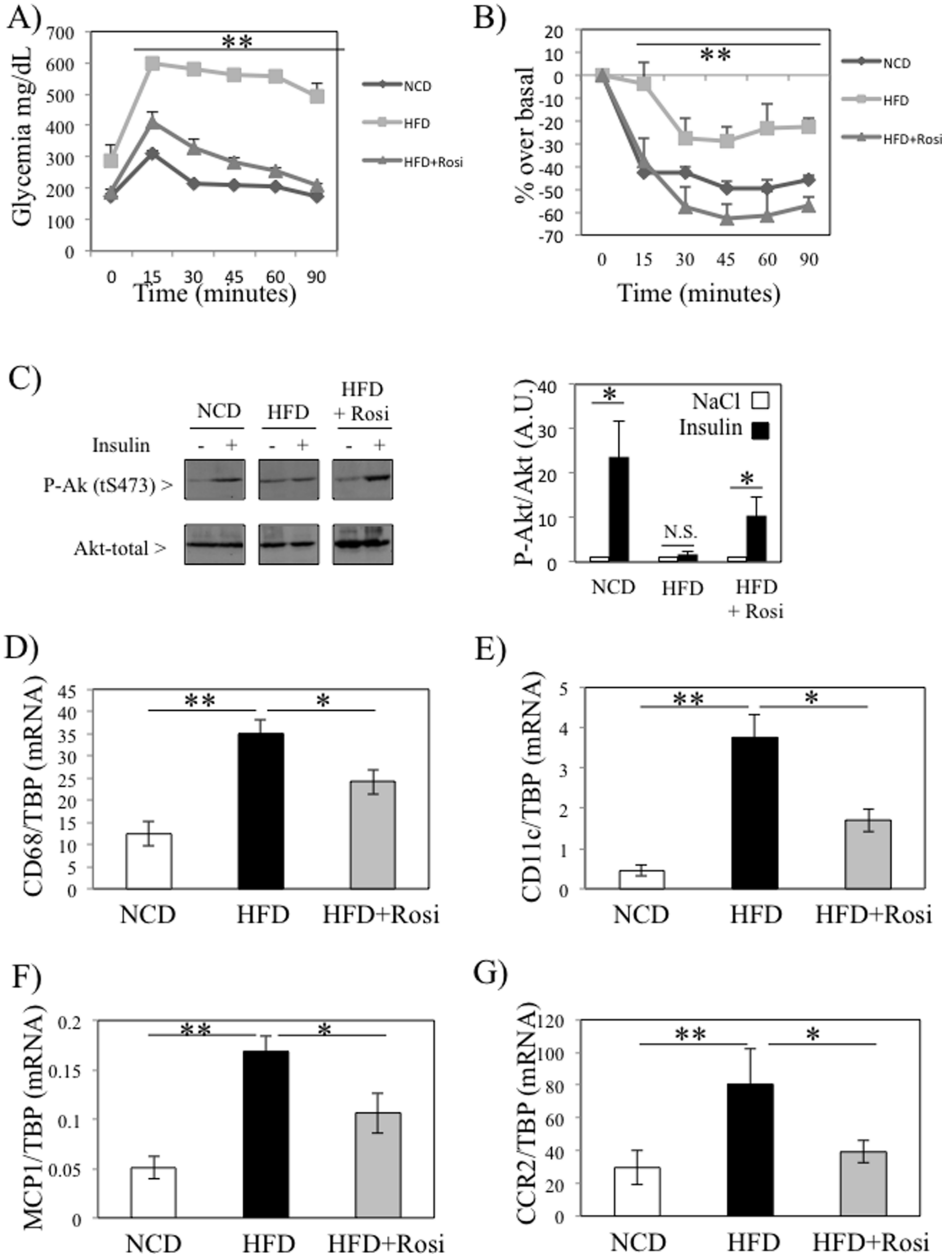
Inflammation is associated with insulin sensitivity in skeletal muscle of diet-induced obese and diabetic mice. A–B) Glucose (A) and insulin (B) tolerance tests were performed on mice on fed a standard diet, a high fat diet (45% Kcal) for 16 weeks in absence or presence of rosiglitazone (200 mg/kg diet) for the last four weeks. Both tests were performed at week 15. C) *Ex-vivo* insulin signalling assay were performed on freshly isolated gastrocnemius. D–G) qPCR analyses of CD68 (D), CD11c (E), MCP1 (F) and CCR2 (G) expressions were performed on quadriceps of mice. * stands for *P*<0.05 and ** for *P*<0.01; n = 5.

We then tested whether inflammation was also increased in skeletal muscles of a nutritional model of diabetes and if the insulin sensitizing agent rosiglitazone could improve this inflammatory state. We hence either fed mice with a normal chow diet (NCD) or with a high-fat diet (HFD, 45% kcal) for 12 weeks, and during the last four weeks a group of HFD mice also received rosiglitazone (200 mg/kg diet) in addition to the HFD. The metabolic characteristics of the mice are shown in Table S3 in File S1. Fasting glycemia and insulinemia, total body weights and both intramuscular and liver TG were increased by the HFD (Table S3 in File S1). Rosiglitazone treatment normalized glycemia, liver TG and decreased circulating FFA levels, whereas total body weights and intramuscular TG were unchanged (Table S3 in File S1). Insulin sensitivity was also evaluated by ipGTT and ipITT, and we demonstrated that HFD caused severe glucose intolerance and insulin resistance, which were reversed by rosiglitazone treatment (Figure 3A and 3B). In addition, insulin-stimulated Akt phosphorylation was altered *ex vivo* in gastrocnemius of HFD mice, and rosiglitazone treatment improved local insulin signaling (Figure 3C). We subsequently isolated mRNA from the quadriceps of these mice and quantified CD68, CD11c, MCP1 and CCR2. The expression of these inflammatory markers was elevated in skeletal muscle of HFD-fed mice, and rosiglitazone treatment prevented these increases (Figure 3D–3G), indicating that skeletal muscle inflammation is closely associated with insulin resistance. However, there were no association between skeletal muscle inflammation and intramuscular triglycerides in these mice (Table S3 in File S1). Lastly, CD68 expression was elevated in skeletal muscles of HFD mice, only after 16 weeks of feeding, whereas no modification was observed after 12 weeks of feeding (Figure S1A). In contrast, CD68 mRNA levels were increased in eWAT of 12 weeks HFD-fed mice (Figure S1B). Interestingly, the elevated expression of CD68 in skeletal muscles of HFD mice correlated with the striking worsening of insulin sensitivity observed between week 11 and week 15, as indicated by the area under the curve (AUC) for the ipGTT and the ipITT (Figure S1C–S1D).

### Palmitate induces macrophages recruitment by C_2_C_12_ muscle cells

**Figure 4:**
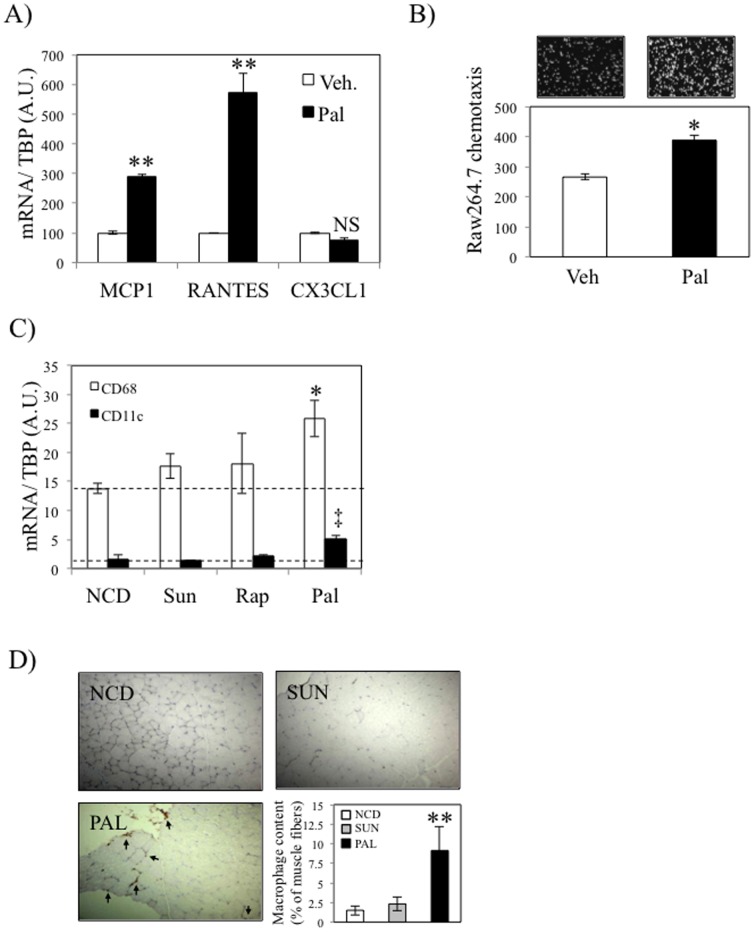
Palmitate induces macrophages recruitment by C_2_C_12_ muscle cells. A) C_2_C_12_ differentiated myotubes were exposed to palmitate (pal, 500 µM) or vehicle (BSA) for 24 hours. MCP1, RANTES and CX3CL1 expression were subsequently determined by real-time PCR. B) Conditioned media from C_2_C_12_ cells treated as indicated in A) were collected and used for chemotaxis assay of Raw264.7 macrophages. Histogram represents the average number of Raw264.7 cells having performed chemotaxis. C) Wild-type C57Bl6 males were either fed a normal chow diet (NCD, low fat) or the same diet enriched with specific lipids sources (20% w/w. Sunflower (Sun), Rapeseed (Rap) and palm oil (Pal). CD11c and CD68 expression in gastrocnemius were subsequently determined by real-time PCR. D) F4/80 immunostaining was performed on muscle sections of mice either fed a normal chow diet, a Sunflower or a Palmitate enriched Diet. Macrophages quantification is shown in the lower right hand panel. In figure 4A, ** stands for *P*<0.01 in palmitate vs vehicle condition. In figure 4C, * stands for *P*<0.05 and ‡ for *P*<0.01 for the respective comparison of CD68 and CD11c between Pal and NCD. NS, not significant; n = 5.

We [9] and others [15], [16] have demonstrated that adipose tissue macrophages (ATMs) are recruited to the eWAT following exposure to excessive concentrations of fatty acids. Furthermore, similar observation was made by Egushi et al. on β-cells in response to palmitate exposure [17]. We hence treated differentiated C_2_C_12_ myotubes with palmitate (500 µM) for 24 hours. Tunel assay experiments did not show any evidence of apoptosis of C_2_C_12_ treated in these conditions (not shown). We then conducted qPCR on the C_2_C_12_ cells and used the resulting conditioned media (CM) for *in vitro* chemotaxis assays of Raw264.7 macrophages. As shown on Figure 4A, palmitate induced the expression of both MCP1 and RANTES in C_2_C_12_, whereas the expression of another chemokine CX3CL1 was not regulated. We also observed that palmitate- treated C_2_C_12_ CM displayed enhanced properties to recruit Raw264.7 macrophages (Figure 4B). As controls, we demonstrated that media alone or containing palmitate had no effect on macrophage recruitment (not shown). The fact that several chemokines such as MCP1 and RANTES were induced, unlike CX3CL1, suggest that Raw264.7 macrophages are recruited in response to the coordinated secretion of an array of chemokines specifically induced in response to palmitate. Finally, in order to verify whether palmitate promotes the recruitment of macrophages *in vivo*, we also quantified the expression of the macrophages markers CD68 and CD11c in gastrocnemius of mice fed a HFD (37.7% Kcal from total lipids, 20% w/w for the indicated lipid) enriched in various fatty acids (sunflower, rapeseed or palm oils) during 8 weeks. The metabolic characteristics of these mice were previously published [4]. Briefly, mice fed different oil-enriched diet showed no modification of body weight and plasma FFA levels. Regarding inflammation, only palm oil enriched diet significant increased of circulating MCP1 and IL6 levels [4]. In this study, we further explored the local inflammation within skeletal muscle of these mice. As shown in Figure 4C, we observed that mice fed a palm oil-enriched diet displayed an increased expression of both CD68 and CD11c in skeletal muscle, whereas sunflower- and rapeseed-enriched diets had no effect on the expression of these markers. In agreement with these observations, palm oil-enriched diet significantly increased macrophages muscle content in mice, whereas sunflower-enriched diet did not modify macrophage infiltration (Figure 4D). The infiltration by macrophages was associated with an increase of TG content in quadriceps of palm oil-enriched diet fed mice compared to NCD mice (1.14±0.14 vs 0.3±0.07 mg/g prot, p = 0.002).

### Skeletal muscle overexpression of MCP1 overexpression induces local inflammation and alters insulin signaling

**Figure 5:**
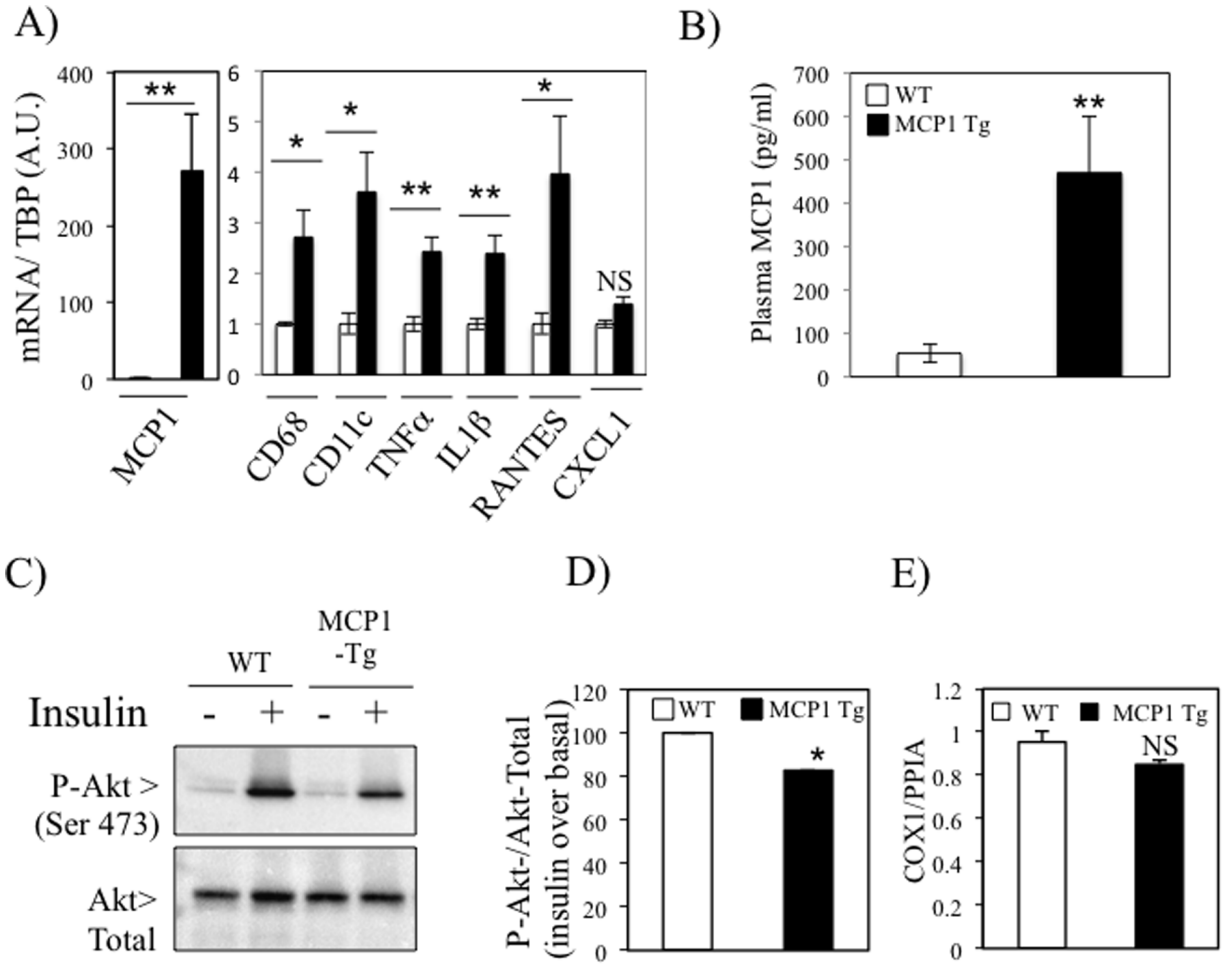
Skeletal muscle overexpression of MCP1 induces local inflammation and alters insulin signaling and glucose metabolism *in vivo*. A) QPCR analyses of MCP1, CD68, CD11c, TNFα, IL1β, RANTES and CXCL1 expression were performed on quadriceps. C–D) *Ex-vivo* insulin signaling assay were performed on freshly isolated gastrocnemius. The histogram (D) represents the fold response to insulin on Akt phosphroylation in Wild-Type (WT) and MCK-MCP1-Tg mice. E) Genomic mitochondrial DNA was evaluated by qPCR in skeletal muscle of WT and transgenic mice. * stands for *P*<0.05 and ** for *P*<0.01; n = 6.

The results obtained on C_2_C_12_ exposed to palmitate suggested that chemokines could mediate the recruitment of macrophages in skeletal muscles in the context of T2D. As MCP1 expression was induced in skeletal muscles of both *ob/ob* and HFD mice (Figures 1D, 2D and 3F), we decided to investigate the role of the chemokine MCP1 in the onset of skeletal muscle inflammation and insulin resistance. We hence generated mice overexpressing MCP1 under the muscle creatin Kinase promoter (MCK). The overexpression of MCP1 in the transgenic mice was confirmed in the quadriceps of the mice at the mRNA level and was also associated with a significant elevation of the macrophages markers CD68 and CD11c as well as the cytokines TNFα and IL1β and the chemokine RANTES (Figure 5A), suggesting that MCP1 overexpression had caused the recruitment of pro-inflammatory macrophages in skeletal muscle tissue. The specific overexpression of MCP1 in skeletal muscle translated in a significant elevation of plasmatic MCP1 levels in transgenic mice (Figure 5B), indicating that MCP1 was released and secreted by MCK expressing muscle cells. Furthermore, we evaluated the local consequences on insulin signaling *ex vivo* and observed that insulin-stimulated Akt phosphorylation was impaired in the gastrocnemius of the MCK-MCP1 trangenic mice (Figures 5C and 5D). These alterations of insulin signaling were observed in absence of modification in the intramuscular TG content (Table S4 in File S1) and the mitochondrial DNA content (Figure 5E) of the MCK-MCP1 transgenic mice.

In order to test whether MCP1 could exert these properties in an autocrine manner on myotubes, we used adenovirus to overexpress MCP1 in differentiated C_2_C_12_. An adenovirus overexpressing GFP (Ad-GFP) was used as control. We validated this construction by showing that adenoviral infection of C_2_C_12_ myotubes elevated the concentration of MCP1 in the conditioned media (Figure S2A). We also used this media to perform chemotaxis assays of Raw364.7 cells. As expected, the CM resulting from C_2_C_12_ infected by Ad-MCP1 had greater chemoattractive properties compared to conditioned media from cells infected with Ad-GFP (Figure S2B). We then tested whether overexpression of MCP1 in C_2_C_12_ altered insulin signaling, and found no direct effect of MCP1 overexpression on insulin-stimulated Akt phosphorylation in C_2_C_12_ cells (Figure S2C). These results indicate that in the absence of resident macrophages, MCP1 overexpression does not alter insulin signaling in a auto or paracrine manner, which is supported by the low expression of CCR2 in the fraction of CD45- cells, containing myotubes, within skeletal muscle (Figure 2B).

### Moderate metabolic alterations in MCK-MCP1 transgenic mice under standard chow diet

**Figure 6:**
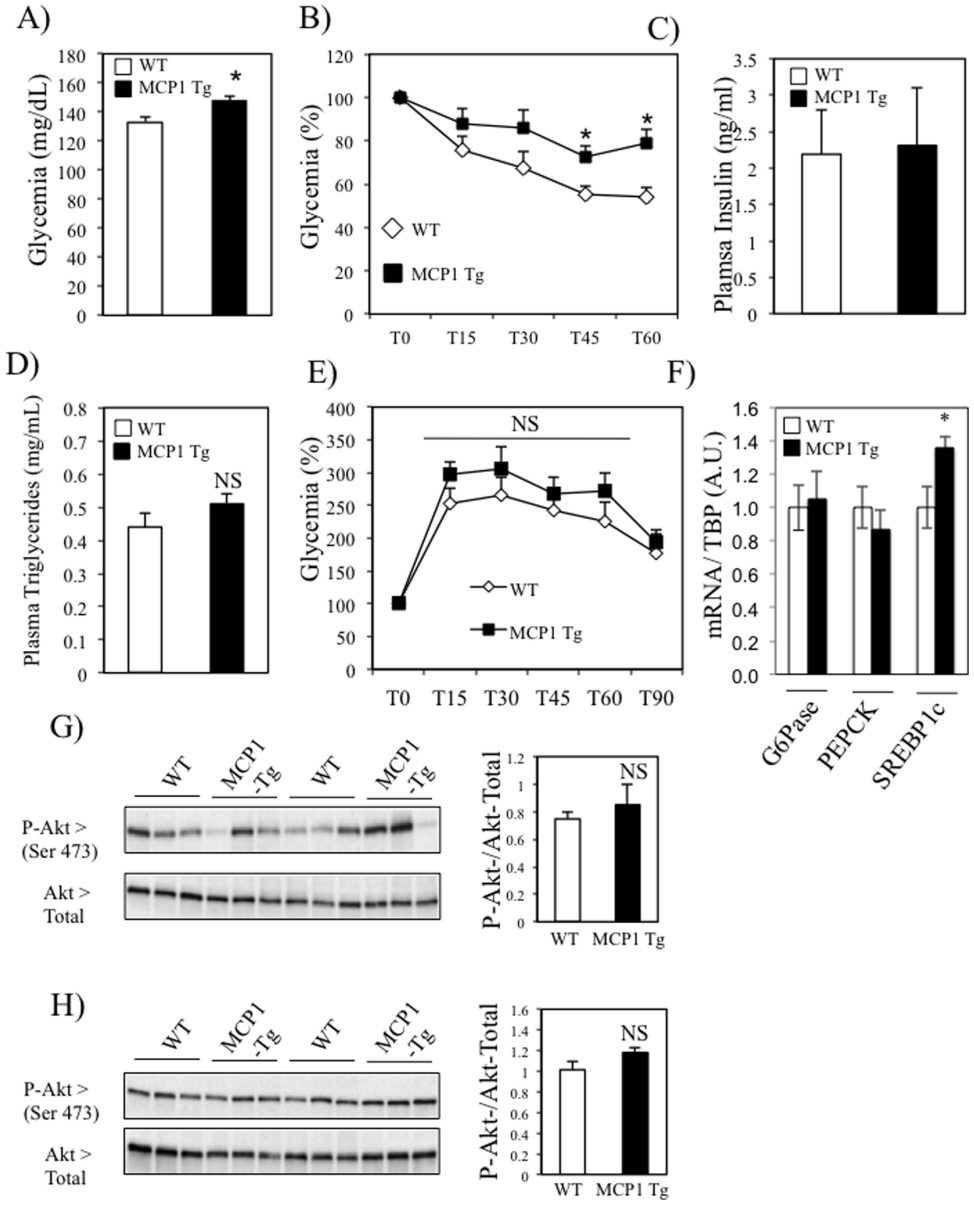
Moderate metabolic alterations in MCK-MCP1 transgenic mice under standard chow diet. A–B) Glycemia was evaluated in basal conditions (A, fed state) and following the insulin injection (ipITT, B). C–D) Fed plasma insulin (C) and TG (D) levels were measured in WT and MCP1- Tg mice E) Evolution of the glycemia during a glucose tolerance test in wt and MCK-MCP1 transgenic mice. F) mRNA quantification of G6Pase, PEPCK and SREBP1c levels in liver of wt and MCK-MCP1 transgenic mice. G–H) Representative images of Western blots (left) and quantification (right) of Serine 473 Akt phosphorylation in liver (G) and adipose tissue (H) of wt and MCK-MCP1 transgenic mice. The histograms on the right of the blots represent the ratio P-Akt (Ser 473)/Akt- total in the liver and the epididymal fat (eWAT) of the mice, respectively.

Skeletal muscle specific overexpression of MCP1 was sufficient to induce local inflammation, and alterations of insulin signaling. We therefore investigated whether this muscle phenotype could impact whole-body glucose homeostasis of mice fed a NCD. As shown in Table S4 in File S1, specific overexpresssion of MCP1 in skeletal muscle of mice did not significantly change the overall body weight of the mice. Furthermore, there was no difference in the weight of the epidydimal fat pads, gastrocnemius, quadriceps, heart and spleen between the two strains (Table S4 in File S1). In addition, we found that MCK-MCP1 transgenic mice had elevated glycemia (Figure 6A) and *ip* insulin tolerance test revealed that these mice showed systemic insulin resistance (Figure 6B). However, the plasma levels of insulin (Figure 6C) and TG (Figure 6D), as well as the glucose clearance (Figure 6E) were not modified in MCK-MCP1 transgenic mice compared to wt mice. As the liver weight was slightly more elevated in the MCK-MCP1 transgenic mice compared to wt mice (Table S4 in File S1), we analyzed this tissue in more details. We found that the expression of the two key gluconeogenic enzymes, G6Pase and PEPCK were not modified in the transgenic mice, whereas the lipogenic transcription factor SREBP1c was more abundantly expressed in the transgenic mice compared to wt (Figure 6F). Additionally, in the fed state there was no significant difference in the phosphorylation level of Akt on Serine 473 in the liver of the two strains (Figure 6G), suggestive of an absence of hepatic insulin resistance in the MCK-MCP1 transgenic mice. Finally, Akt phosphorylation level was also unaltered in the epididymal fat of the MCK-MCP1 transgenic mice (Figure 6H). Altogether, these data suggest that both the liver and the white adipose tissue are unlikely to participate in the moderate worsening of glucose homeostasis observed in the MCK-MCP1 transgenic mice fed a NCD.

### Inflammation markers are increased in skeletal muscles of type 2 diabetic patients and correlate with HOMA-IR

**Figure 7:**
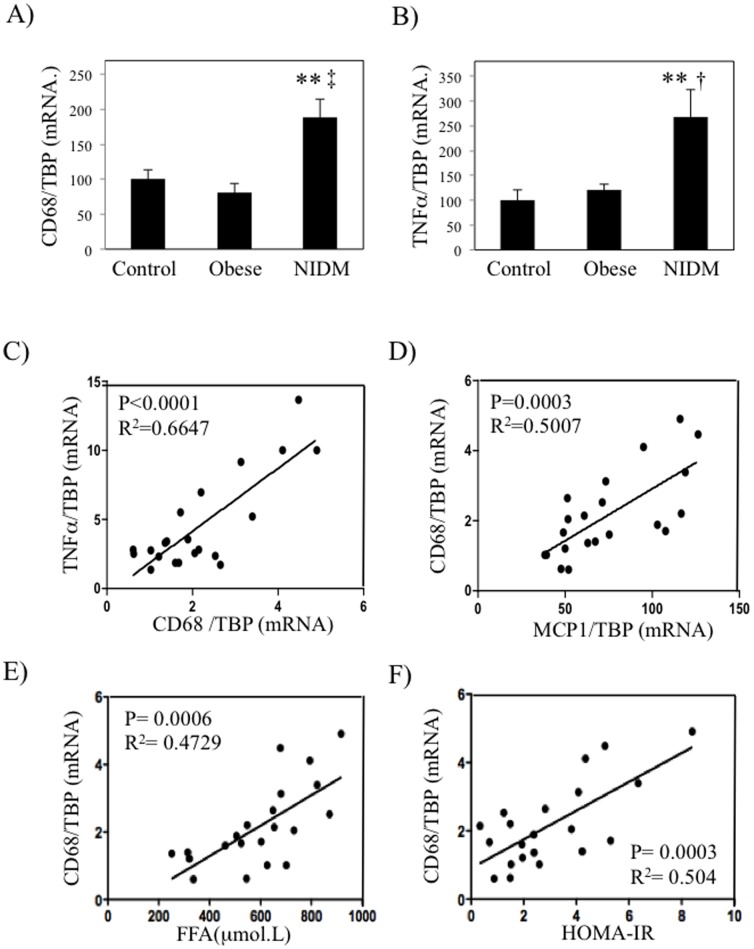
Inflammation markers are increased in skeletal muscles of type 2 diabetic patients and correlate with HOMA-IR. Biopsies from vastus lateralis of control subjects (n = 8), obese non-diabetic patients (n = 9) and obese type 2 diabetic patients (NIDM, Non Insulin-dependent Diabetes Mellitus) (n = 10) were collected and qPCR were performed for CD68 (A) and TNFα (B). Correlation analyses between the expression of CD68 and TNFα (C) and MCP1 and CD68 in the vastus lateralis of patients (D). E–F) Correlation analyses between plasma FFA and HOMA-IR levels of subjects and CD68 expression, respectively. ** stands for *P*<0.01 when comparing NIDM group with Control, † stands for *P*<0.05 and ‡ for *P*<0.01 when comparing NIDM with Obese.

Our results obtained in murine models prompted us to test whether similar associations could be observed in human. We therefore collected biopsies from vastus lateralis of lean, obese healthy and obese type 2 diabetic patients (clinical and metabolic characteristics are shown in Table 1. Fasting glycemia, but not insulinemia (p = 0.06), was significantly more elevated in type 2 diabetic patients compared to both lean and obese subjects. However, both fasting glycemia and insulinemia, as well as HOMA-IR, were not significantly increased in obese subjects compared to lean subjects. Nevertheless, obese subjects presented a clear trend towards higher insulinemia and HOMA-IR, which was indicative of an insulin resistance state of this population. Furthermore, plasma FFA levels were more elevated in T2D compared to obese patients. The mRNA levels of CD68 and TNFα were significantly upregulated only in muscle of type 2 diabetic patients but not in weight matched insulin sensitive or lean patients (Figure 7A and 7B). Interestingly, CD68 mRNA levels were correlated with TNFα mRNA levels (Figure 7C), suggesting that local macrophages were responsible for the expression of this cytokine in skeletal muscles. In agreement with our previous observations made in mice and *in vitro*, CD68 expression in vastus lateralis was significantly associated with MCP1 expression in skeletal muscles (Figure 7D). We also determined the correlation between CD68 expression and HOMA-IR of these subjects. As shown in Figure 7E and 7F, the expression of CD68 was significantly correlated with patients plasma FFA levels and the degree of insulin resistance of the patients.

## Discussion

WAT macrophage infiltration is generally elevated in T2D and results in an increase of pro-inflammatory cytokines levels that participate to local insulin resistance. Whereas local inflammation is also increased in skeletal muscle of both diabetic mice and T2D patients, its exact contribution to the development of insulin resistance is subject to debate [18]. In the present study, we demonstrated that both the expression of inflammatory markers and the macrophage infiltration are increased in several mice models of T2D, extending beyond the high fat diet model. In particular, in this study we characterized the skeletal muscle macrophages populations in *ob/ob* mice, and demonstrate that pro-inflammatory macrophages markers are increased whereas anti-inflammatory macrophages markers are decreased in this tissue. Interestingly, we bring evidence that the expression of the cytokine TNFα in skeletal muscle tissue can very largely be attributed to myeloid cells within skeletal muscle tissue, whereas the cytokine IL6 is mostly expressed by non-myeloid cells within the tissue. We further bring evidences that these macrophages could be recruited in response to the concerted up regulation of chemokines, including MCP1 whose expression in myotubes is more elevated upon exposure to excessive concentrations of palmitate. Interestingly, skeletal muscle specific overexpression of MCP1 in transgenic mice was able to induce local inflammation and local alteration of insulin signaling under a NCD. A possible paracrine alteration of MCP1 on insulin signaling was excluded as MCP1 overexpression in myotubes did not have any consequences on this pathway, which was consistent with the very low expression of CCR2 in muscle cells within skeletal muscle tissue. In addition, we found a moderate repercussion of muscle MCP1 overexpression on whole-body glucose homeostasis under NCD. However, in absence of full phenotypic characterization of the transgenic mice, we cannot exclude that this modest modification of whole-body glucose homeostasis was not related to other potential confounding factors secondary to MCP1 overexpression, such as differential food intake, spontaneous physical activity or muscle wasting between the WT and the MCK- MCP1 transgenic mice. Finally, we demonstrate that the expression of the macrophage markers CD68 and TNFα is more elevated in skeletal muscle of type 2 diabetic patients than in both lean healthy subjects and BMI-matched obese non- diabetic patients, and CD68 expression correlated with both circulating FFA levels and patients insulin sensitivity. Altogether, our data suggest that local inflammation within skeletal muscle could participate to the development of insulin resistance.

The fact that macrophage infiltration occurs in skeletal muscle in the context of T2D was previously suggested by other elements. Firstly, plasmatic concentrations of cytokines in rodent models of T2D are lower than concentrations used to alter insulin signaling *in vitro* in myotubes [19], [20]. Secondly, when reporter mice for the activity of NF-κB were fed a HFD, the existence of local inflammation sites were not only seen in WAT but also in kidney, liver and skeletal muscle [21]. Thirdly, increased inflammation was observed in skeletal muscles of murine models of T2D but also in obese subjects where pro-inflammatory macrophages infiltration correlated with patients BMI and age [22], [23]. However, the infiltration of macrophages within muscle was reported to be low compared to WAT [24]. Our present study describes muscle inflammation and macrophage accumulation in four different murine models and in type 2 diabetic patients. In these patients, we demonstrated that inflammation in vastus lateralis muscle was correlated with insulin resistance independently of subject BMI. In addition, treatment of HFD mice with Rosiglitazone decreased muscle inflammation concomitantly with local insulin signalling improvement. Interestingly, the lower skeletal muscle inflammation observed in response to rosiglitazone administration was supportive of the notion that skeletal muscle triglycerides content and insulin resistance can be disconnected, such as seen in several models [25]–[27]. This situation is also observed in the MCK-MCP1 transgenic mice where alteration of insulin signaling is observed without any change in the intramuscular TG content. Consequently, in these models skeletal muscle inflammation is better associated with local insulin resistance than skeletal muscle triglycerides. Whereas, we cannot exclude a contribution of reduced WAT inflammation and lower plasma cytokines levels in the local improvement of insulin signaling within muscle of rosiglitazone-treated HFD mice, the fact that the improved phenotype is conserved *ex-vivo* in muscle explants (as described in Figure 3C), favors the hypothesis of a closed relationship between local macrophage infiltration and muscle insulin sensitivity.

Our observations made in HFD mice suggest that muscle macrophages infiltration is a rather late event in T2D etiology. This observation is in agreement with the fact that fatty acids are needed to stimulate the secretion of macrophages chemoattractants, such as MCP1, to further induce the recuitment of macrophages in skeletal muscles. *In vivo*, increased FFA delivery towards skeletal muscle occurs when WAT becomes insulin resistant, further causing a rise in the flux of FFA delivered to other organs. In consequence, while WAT macrophages may promote early events in the development of diabetes, skeletal muscle macrophages may instead contribute to late stages of evolution of the disease, consistently with the adipo-centric models of diabetes etiology [28]–[30]. Recently, the infiltration of pro-inflammatory macrophages in association with obesity was also described in the pancreas, and was shown to contribute to β cells dysfunction by activating inflammatory processes in the islets [17]. Consequently, macrophage recruitement seems to occur in several tissues in T2D context, suggesting a general state of inflammation rather than a WAT-restricted inflammation.

Macrophage infiltration is reportedly massive in models where a lesion is induced in skeletal muscles. In these models, the contribution of the axis MCP1/CCR2 in governing the recruitment of macrophages to skeletal muscles is well established [14], [31]. In these contexts, macrophages are involved in diverse functions extending well beyond the stimulation of inflammation and as such they influence tissue repair, myogenesis and angiogenesis. Interestingly, in the present study, we observed an overexpression of MCP1 at both circulating and intramuscular levels in all mice models of obesity and insulin resistance tested. This upregulation is similar to what has been observed in the WAT [32], [33] and suggest that MCP1 responsive, i.e. CCR2+ macrophages, are recruited to skeletal muscles, where they further worsen insulin resistance. However, in the case of the WAT several other chemokines seem to be involved in the recruitment of macrophages [34]–[36]. This situation may be similar in the skeletal muscle as for instance RANTES, another macrophage chemokine, is also upregulated in skeletal muscle of both HFD and *ob/ob* mice (not shown). In addition, Bouzakri et al [19] demonstrated that differentiated human myotubes secrete numerous chemokines, which may serve as myokines. Expression of these chemokines is regulated by physical activity but also exposure to cytokines such as TNFα. We report here that FFA is a modulator of MCP1 secretion and potentially of others myokines. Furthermore, the role of fatty acids in mediating the detrimental properties of inflammatory cells may indirectly be exemplified by the partial alteration of glucose metabolism observed in MCK-MCP1 transgenic mice fed a standard diet. Indeed, a similar mild phenotype was observed in adipose MCP1 transgenic mice fed a standard diet, despite elevated local adipose inflammation. Hence, it is plausible that similar to the adipose MCP1 transgenic mice, the MCK-MCP1 mice require an additional challenge represented by a high fat diet, in order to amplify their metabolic phenotype [32], [33]. Finally, very recently Pedersen et al demonstrated that the overexpression of the chemokine CXCL1 attenuated diet-induced obesity and improved fatty acid oxidation in muscles [37]. Our work suggests that these chemokines may not act directly on myotubes but instead act indirectly in governing the recruitment of different subsets of leukocytes, which is in agreement with the expression of the receptor for MCP1, CCR2, which is barely detected by CD45- cells within skeletal muscles.

Elevation of intramuscular inflammation is not only observed in T2D since it is also a hallmark feature of both aging and sarcopenia [38]. Sarcopenia is itself associated with aging and worsened by T2D and inflammation [39]. Interestingly, TNFα is a pro-inflammatory cytokine that not only promotes insulin resistance [29], [40], it also induces sarcopenia [41]. In the present study, we observed that within skeletal muscle, TNFα expression is almost exclusive to CD45+ cells, which is comparable to the situation in the WAT, where TNFα expression is more prevalent in the stromal vascular fraction [13]. This specific restriction in the expression of TNFα clearly indicates that this population of cells should further be studied as they may represent attractive targets in the treatment of T2D and potentially as well other conditions associated with aging such as sarcopenia.

In conclusion, our data clearly indicate that insulin resistance is associated with skeletal muscle inflammation in both mice and humans, and suggest that lipid-induced local secretion of MCP1 participate to the recruitment of macrophages within muscle, and to subsequent alterations of muscle insulin signaling. Therefore, our study suggests that the modulation of muscle inflammation may provide an exciting new avenue for improving insulin resistance.

## Supporting Information

Figure S1 Epididymal white adipose adipose tissue (eWAT, A) and quadriceps (B) were collected at week 12 and week 16 of the protocol of CD68 expression quantified with QPCR. Area Under the Curves (AUC) corresponding to the ipGTT (C) and ipITT (D) perfomed at week 3, 8, 11 and 15 after initiating the dietary intervention (NCD, Normal Chow Diet, HFD, High Fat Diet (45%Kcal). ** stands for *P*<0.01. NS, not significant; n = 5.
(TIFF)

Figure S2 A) C_2_C_12_ differentiated myotubes were infected with adenovirus overexpressing either GFP or MCP1 for 24 hours (Ad-GFP and Ad-MCP1, respectively) and MCP1 concentration was subsequently determined by ELISA in the conditioned media. B) Conditioned media of infected C_2_C_12_ were also used to perform chemotaxis assays on Raw264.7 macrophages. C) Akt phospshorylation (Ser 473) was measured by western-blot in order to evaluate insulin signaling in C2C12 myotubes following adenoviral mediated overexpression of MCP1. ** stands for *P*<0.01.
(TIFF)

File S1 Supporting Tables S1, S2, S3 and S4.
(DOC)
