# Supplementary figures and images for: Insulin Resistance is Associated with MCP1-Mediated Macrophage Accumulation in Skeletal Muscle in Mice and Humans
Source: PLoS One. 2014 Oct 22;9(10):e110653. doi: 10.1371/journal.pone.0110653 (PMC4206428; doi:10.1371/journal.pone.0110653)

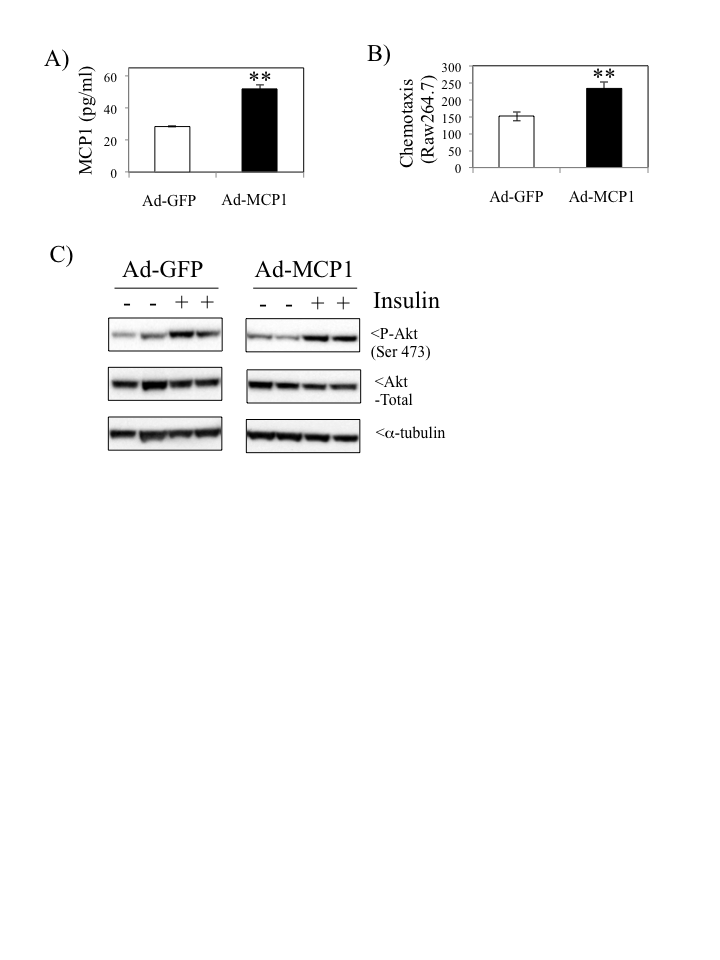

Supplement: Figure S2 — A) C2C12 differentiated myotubes were infected with adenovirus overexpressing either GFP or MCP1 for 24 hours (Ad-GFP and Ad-MCP1, respectively) and MCP1 concentration was subsequently determined by ELISA in the conditioned media. B) Conditioned media of infected C2C12 were also used to perform chemotaxis assays on Raw264.7 macrophages. C) Akt phospshorylation (Ser 473) was measured by western-blot in order to evaluate insulin signaling in C2C12 myotubes following adenoviral mediated overexpression of MCP1. ** stands for P<0.01. (TIFF) [file pone.0110653.s002.tiff]
