# Supplementary material for: Insulin Resistance is Associated with MCP1-Mediated Macrophage Accumulation in Skeletal Muscle in Mice and Humans
Source: PLoS One. 2014 Oct 22;9(10):e110653. doi: 10.1371/journal.pone.0110653 (PMC4206428; doi:10.1371/journal.pone.0110653)
Supplement: File S1 — Supporting Tables S1, S2, S3 and S4. (DOC) [file pone.0110653.s003.doc]

**Table S1: Primers sequences used for QPCR**

| **species** | **gene** | **sequence (5'-3')** |
| --- | --- | --- |
| mouse | **CCR2 F** | GCC-AGG-ACA-GTT-ACC-TTT-GG |
|  | **CCR2 R** | CGA-AAC-AGG-GTG-TGG-AGA-AT |
| mouse | **CD4 F** | GAG-AGT-CAG-CGG-AGT-TCT-C |
|  | **CD4 R** | CTC-ACA-GGT-CAA-AGT-ATT-GTT-G |
| mouse | **CD8a F** | CAG-AGA-CCA-GAA-GAT-TGT-CG |
|  | **CD8a R** | TGA-TCA-AGG-ACA-GCA-GAA-GG |
| mouse | **CD11c F** | ACA-CAG-TGT-GCT-CCA-GTA-TGA |
|  | **CD11c R** | GCC-CAG-GGA-TAT-GTT-GAC-AGC |
| human | **CD68 F** | TCA-GCT-TTG-GAT-TCA-TGC-AG |
|  | **CD68 R** | AGG-TGG-ACA-GCT-GGT-GAA-AG |
| mouse | **CD68 F** | TCA-GCT-AAA-CTC-GCT-CAA-TC |
|  | **CD68 R** | TCC-AGC-CTG-TTG-TAA-CTG-AG |
| mouse | **FOXp3 F** | ACT-CGC-ATG-TTC-GCC-TAC-TTC-AG |
|  | **FOXp3 R** | GGC-GGA-TGG-CAT-TCT-TCC-AGG-T |
| mouse | **IL1β F** | ACT-GTT-CCT-GAA-CTC-AAC-TG |
|  | **IL1β R** | CTT-GTT-GAT-GTG-CTG-CTG-CG |
| human | **MCP1 F** | TCA-GCC-AGA-TGC-AAT-CAA-TG |
|  | **MCP1 R** | ATG-GTC-TTG-AAG-ATC-ACA-GC |
| mouse | **MCP1 F** | TGG-AGC-ATC-CAC-GTG-TTG-GC |
|  | **MCP1 R** | ACT-ACA-GCT-TCT-TTG-GGA-CA |
| mouse | **MYOG F** | CAA-CCC-AGG-AGA-TCA-TTT-GC |
|  | **MYOG R** | CAT-ATC-CTC-CAC-CGT-GAT-GC |
| mouse | **TBP F** | TGG-TGT-GCA-CAG-GAG-CCA-AG |
|  | **TBP R** | TTC-ACA-TCA-CAG-CTC-CCC-AC |
| mouse | **TLR4 F** | AGT-GGC-TGG-ATT-TAT-CCA-GG |
|  | **TLR4 R** | TCC-ACA-GCC-ACC-AGA-TTC-TC |
| human | **TNFα F** | AGC-CCA-TGT-TGT-AGC-AAA-CC |
|  | **TNFα R** | GAG-GTA-CAG-GCC-CTC-TGA-TG |
| mouse | **TNFα F** | CCA-GAC-CCT-CAC-ACT-CAG-ATC |
|  | **TNFα R** | CAC-TTG-GTG-GTT-TGC-TAC-GAC |

**Table S2: Metabolic characteristics of wt and ob/ob mice (n=5) (6 hours fasted). FBG, Fasting Blood Glucose * p<0.05, ** p<0.01, *** p<0,001.**

|  | WT | ob/ob |
| --- | --- | --- |
| body weight (g) | 26.7 ± 0,2 | 55.9 ± 0,2 ** |
| plasma TNFα (pg/ml) | 18.4 ± 3,5 | 33.4 ± 7.8 ** |
| plasma IL10 (pg/ml) | 38 ± 2.8 | 39 ± 3.5 |
| FBG (mmol/l) | 9.5 ± 0.9 | 15.3 ± 1.8 ** |
| Insulin (pmol/l) | 56.8 ± 17.2 | 2.92 ± 344 ** |
| Plasma TG (g/l) | 0.37 ± 0,04 | 0.54 ± 0.07 * |
| quadriceps TG content (mg/g prot) | 0.04 ± 0.01 | 1.61 ± 0.07*** |

**Table S3: Metabolic characteristics of NCD, HFD ± Rosi mice (n=10).**

NCD: normal chow diet, HFD: high fat diet, Rosi: Rosiglitazone, TG: triglyceride, FFA: free fatty acids.

** p<0.01 HFD Vs NCD; † ‡ p<0.01 HFD+Rosi Vs NCD, ¥ p<0.05 HFD + Rosi Vs HFD; NS: Not Significant.

|  | **NCD** | **HFD** | **HFD + Rosi** |
| --- | --- | --- | --- |
| body weight (g) | 27.9 ± 1.2 | 45.9 ± 2(**) | 46.8 ± 1.7 (‡) |
| glycemia (mg/dl) | 174 ± 8.8 | 289 ± 50.1(*) | 189 ± 6.5 (¥) |
| Insulin (ng/ml) | 0.31 ± 0.08 | 1.83 ± 0.49(**) | 1.25 ± 0.48 (†) |
| plasma TG (g/l) | 0.61 ± 0.1 | 0.57 ± 0.06 (NS) | 0.40 ± 0.04 (†) |
| plasma FFA (mmol/l) | 0.63 ± 0.09 | 0.73 ± 0.08 (NS) | 0.50 ± 0.08 (¥) |
| liver TG (mg/mg Prot) | 0.15 ± 0.01 | 0.43 ± 0.08 (*) | 0.15 ± 0.02 (¥) |
| quadriceps TG (mg/g Prot) | 0.63 ± 0.16 | 1.61 ± 0.19 (**) | 1.88 ± 0.16 (‡) |

**Table S4: Characteristics of wild-type Vs MCK-MCP1 transgenic mice (n=6)**. eWAT, epididymal white adipose tissue, TG : triglyceride.

|  | **mean** | | **sem** | | **p value** |
| --- | --- | --- | --- | --- | --- |
| Genotype | **Wild-type** | **MCP1-Tg** | **Wild-type** | **MCP1-Tg** |  |
| Body Weight (g) | 28.27 | 31.28 | 1.24 | 0.70 | 0.06 |
| Liver weight (g) | 1.35 | 1.53 | 0.06 | 0.04 | 0.02 |
| Quadriceps weight (g) | 0.35 | 0.38 | 0.02 | 0.01 | 0.27 |
| Gastrocnemius weight (g) | 0.30 | 0.32 | 0.01 | 0.01 | 0.11 |
| Spleen weight (g) | 0.09 | 0.11 | 0.00 | 0.01 | 0.11 |
| eWAT weight (g) | 0.58 | 0.71 | 0.10 | 0.14 | 0.46 |
| Heart weight (g) | 0.15 | 0.15 | 0.01 | 0.01 | 0.86 |
| quadriceps TG content (mg/g Prot) | 0.14 | 0.20 | 0.02 | 0.04 | 0.29 |
